# Impact of *DRD2/ANKK1* and *COMT* Polymorphisms on Attention and Cognitive Functions in Schizophrenia

**DOI:** 10.1371/journal.pone.0170147

**Published:** 2017-01-13

**Authors:** Irene Nkam, Nicolas Ramoz, Florence Breton, Jasmina Mallet, Philip Gorwood, Caroline Dubertret

**Affiliations:** 1 INSERM U894, Center of Psychiatry and Neurosciences, Paris, France; 2 Pôle 1, Roger Prevot Hospital, Moisselles, France; 3 Paris Descartes University, Sorbonne Paris Cite, Faculty of Medicine, Paris, France; 4 AP-HP, Department of Psychiatry, Louis Mourier Hospital, Colombes, France; 5 Paris Diderot University, Sorbonne Paris Cite, Faculty of Medicine, Paris, France; 6 Clinique des maladies mentales et de l’encéphale, Sainte-Anne Hospital, Paris, France; University of Texas Health Science Center at San Antonio Cancer Therapy and Research Center at Houston, UNITED STATES

## Abstract

**Background:**

Cognitive deficits such as poor selective attention and executive functions decline have been reported in patients with schizophrenia. Many studies have emphasized the role of dopamine in regulating cognitive functions in the general population as well as in schizophrenia. However, the relationship between cognitive processes, schizophrenia and dopaminergic candidate genes is an original approach given interesting results. The purpose of the current exploratory study was to examine the interaction of dopaminergic genes (coding for dopamine receptor D2, *DRD2*, and for Catecholamine-O-Methyl-Transferase, *COMT*) with the diagnostic of schizophrenia in (i) the executive control of attention, (ii) selective attention, and (iii) executive functions.

**Methods and Results:**

We recruited 52 patients with schizophrenia and 53 healthy controls who performed the Stroop Color-Word Test, the Attention Network Test and the Wisconsin Card Sorting test. Four single nucleotide polymorphisms (SNPs) in the *DRD2* gene (rs6275, rs6277, rs2242592 and rs1800497) and two SNPs in the *COMT* gene (rs4680 and rs165599) have been genotyped. Patients with schizophrenia performed significantly worse than controls in all cognitive performance, taking into account demographic variables. A significant gene by disease interaction was found for the Stroop interference (p = 0.002) for rs6275 of the *DRD2* gene. The *COMT* Val/Val genotype and schizophrenia were associated with increased number of perseverative errors (p = 0.01).

**Conclusions:**

In our study, the *DRD2* gene is involved in attention while the *COMT* gene is implicated in executive functions in patients with schizophrenia.

## Introduction

Schizophrenia is a complex and severe disorder affecting 1% of the population, with high heritability, estimated around 80%, suggesting a strong involvement of genetic factors [1]. Many genes are reported to be associated with the disease, each conferring only a small risk [2, 3]. Environmental factors and interactions between genetic and environment are also likely to contribute to schizophrenia [4, 5]. Dopamine system has been the focus of decades of research in schizophrenia. The gene coding for the dopamine receptor D2 (*DRD2)*, located on chromosome 11q22-2, is one of the most studied marker of susceptibility for schizophrenia [6]. We have previously reported a strong association between schizophrenia and various polymorphisms within the *DRD2/ANKK1* locus, including rs1800497 in the *ANKK1* gene, including rs2242592 in the intergenic region and rs6277 in *DRD2* gene [7]. Moreover, rs1800497 was over-transmitted from parents to the affected child [8]. Genetic studies have confirmed the positive association between rs1800497 and schizophrenia (for review, we refer the reader to [9]).

In spite of some controversies, alterations in catecholamine metabolism have also been studied in schizophrenia [10, 11]. Catecholamine-O-Methyl-Transferase (*COMT)* gene located in chromosome 22q11 codes for an enzyme that degrades catecholamines, including dopamine [10]. The *COMT* Val^108/158^Met polymorphism (rs4680) influences enzyme activity, with the valine allele associated with a higher enzymatic activity and an increase risk of schizophrenia [10, 12]. The G allele of the rs165599 in *COMT* has also been associated with schizophrenia [13].

Neuropsychological impairments have been reported in schizophrenia and could be present from the first psychotic episode [14, 15]. Cognitive deficits in (i) the executive control of attention, (ii) selective attention, and (iii) executive functions are considered to be core features of the disease and have an important role on the prognosis and functional disability associated with schizophrenia [16, 17]. Their poor performances may portend worst outcome [15]. One of the most commonly observed cognitive disturbances in schizophrenia patients is deficit in performance on task in selective attention [18]. The executive control of attention governs the capacity to decide among conflicting responses and to give a response to one aspect of a stimulus by ignoring a dominant aspect. The executive control of attention is often studied by tasks that involve conflict such as the Stroop Color-Word. Using this paradigm, schizophrenia is associated with a reduction in the ability of cognitive inhibition of an overlearned response [for review [19]. In healthy controls, the attention and the conflict process activate the anterior cingulate cortex [20].

Attention is the basis of various control systems, and is defined as the efficiency of three reliable, anatomically-distinct organized networks: alerting, orienting and executive control [21]. The attention network test (ANT) is a paradigm designed to investigate attention efficiencies of alerting, orienting, and executive control of attention [22], on the basis of a concept of an integrative selective attention system [23]. The alerting function shows strong thalamic involvement and activation of fronto-parietal cortex [24], and the orienting function activates parietal lobe [25]. To assess executive function, the Wisconsin Card Sorting Test (WCST) is commonly used. It has been shown that the performance of the WCST engages the prefrontal cortex in healthy volunteers [26, 27] and in schizophrenia patients [28].

Attentional performances in the general population are influenced by genetic factors. Fan et al. [29] showed that genetic variations, contributing to individual differences in the executive attention of the ANT performance, involve dopamine rich frontal areas, including the anterior cingulate. An interaction effect between rs4680 *COMT* gene and rs1800497 *ANKK1/DRD2* locus on interference performance on the Stroop Color-Word test has been found in a sample from the general population. A worsened Stroop performance has been observed in subjects homozygous for the Met allele at rs4680 in the absence of T allele of rs1800497 [30]. Healthy subjects carrying CC genotype of the rs1800497 have revealed a significant greater conflict when they performed the ANT protocol [31]. A previous study has also shown a slight statistical trend toward higher conflict ANT for healthy subjects homozygous Met/Met of the *COMT* gene [32]. However, this observation was not replicated [33]. The executive components of attention may be heritable in normal subjects.

Thus, the Stroop Color-Word test as the executive control of the ANT serve as a possible endophenotype in schizophrenia [29, 34, 35]. Less is known about the association between schizophrenia, the executive control of attention and genetic factors.

Concerning executive performances, Rodriguez-Jimenez et al. have found in healthy subjects that the carriers of the T allele of the rs6277 performed better at WCST than carriers of the CC genotype [36]. Finally, CC genotype of rs6277 has been associated with a decreased of the general cognitive ability, while no difference has been found with rs4680 of the *COMT* gene [37].

The present exploratory study has investigated four SNPs in the *DRD2/ANKK1* locus (rs6275, rs6277, rs2242592 and rs1800497) and two SNPs in the *COMT* gene (rs4680 and rs165599) in order to analyze their association with attention and executive function, in patients with schizophrenia as compared to healthy volunteers. For each SNP, the allele or the genotype previously associated to schizophrenia and considered as the “vulnerability” allele or genotype according to literature has been compared between patients and controls. The aim of the current study was (a) to test the hypothesis that patients carrying the vulnerability allele or genotype of each SNP show a decline in cognitive performances compared with subjects homozygous for the other allele and (b) to examine the possible interactions between disease and SNPs influences on cognitive efficiency.

## Materials and Methods

### Subjects

We recruited 52 outpatients with schizophrenia and 53 healthy controls from a psychiatric department of a French university hospital in Paris suburb. All subjects were evaluated by an experienced psychiatrist using the Diagnostic Interview for Genetic Studies (DIGS) [38], that is a semi-structured interview leading to a lifetime diagnosis according to the DSM-IV criteria for schizophrenia and other psychiatric diseases [39]. Inclusion for patients with schizophrenia required a combination of criteria including (1) having a stable state with no change in medication or symptoms for at least 4 weeks before the cognitive evaluation, (2) being treated exclusively with atypical antipsychotics, with no anticholinergic agents or mood stabilizers, (3) having had a relatively short course of illness (less than 10 years). All patients are clinically stable, as assessed with the PANSS [40], and fully able to cooperate with testing. According to the literature, a relatively short illness follow-up time and all being treated with atypical antipsychotics are supposed to have beneficial effect on cognition [41, 42] with no other drugs that affect cognition, allowing to study a homogeneous group of patients.

Controls were included only if they were between 28 and 65 years old and were recruited through partners of patients admitted in the psychiatric department. Control subjects had no first-degree family history of bipolar or schizophrenia disorder. All subjects (patients and controls) were without any history of current substance abuse, brain injury or neurological disease, medical condition or medication known to be associated with neuropsychological impairment. All participants had normal or corrected-to-normal vision. All subjects were euthymic as evaluated by the Montgomery and Asberg Rating Scale (MADRS) [43] and the Young Mania Rating Scale [44], and they had no mental retardation as evaluated by National Adult Reading Test (NART) [45].

Patients and healthy volunteers all gave their written informed consent after hearing a complete description of the study. The study protocol was approved by the French Ethics and Data Protection and Freedom of Information Commissions (Comité Consultatif National d’Ethique and the Commission Nationale d‘Informatique et des Libertés).

### Procedure

Patients and healthy controls have performed the Stroop Color-Word Test, the Attention Network Test, and the Wisconsin Card Sorting Test to assess attention and executive functions.

The Stroop Color-Word interference test measures selective attention [46]. This task tests the ability to attend only to the color in which a word is written while ignoring the distractor, which is what the word means. Participants are presented with a series of cards containing 100 stimuli from one condition and are asked to respond to each stimulus without stopping. The total time per card is the measure of performance in a given condition. The subject is asked first to read the names of colors written in black ink (first condition), then is asked to name the color of non-color words written in colored ink (second condition). The third condition elicits what is called the Stroop interference effect, within the subject must name the color of the ink in which the names of various colors are written while ignoring the word. We determined the Stroop Color-Word interference score (Stroop-PI), which is latency for naming all of the colors correctly in this third condition, minus the time required for the second condition.

The ANT is a recent paradigm designed to investigate efficiencies of alerting, orienting and executive control of attention [22]. The stimuli consist of a row of five visually-presented, white lines, with arrowheads pointing leftward or rightward, against a black background; the target is a leftward or rightward arrowhead at the center. This target is flanked on either side by two arrows pointing in the same direction (congruent condition), by two arrows in the opposite direction (incongruent condition), or by 2 lines (neutral condition). The participant’s task is to indicate the direction of the central target by right- or left- clicking the mouse as quickly as possible. Cues console consists of a 100 ms asterisk presented 400 ms before the target. There were four cue conditions: no cue, central cue which appear at the central fixation point, double-cue in which two warming cues correspond to two possible target positions above and below the central fixation point, and spatial cue which was presented on the exact target localization. The executive effect was calculated by subtracting the mean reaction times (RT) of the condition with congruent flankers from the mean RT of the condition with incongruent flankers. We determine the executive control of attention (ANTc), the median reaction time (ANT-RT), and the percentage of correct answers (ANT%).

The Wisconsin Card Sorting Test (WCST) was used to assess executive processes [47, 48]. This task includes four stimulus cards and 128 response cards that contain various figures. The cards vary for color (red, yellow, blue, green), type of figure (crosses, circles, triangles or stars), and number of figures (one to four). The test was discontinued after the completion of six categories or when no more response card remained. The chosen variables were the numbers of perseverative errors (WCST-PE), which reflects the tendency towards perseveration, and the number of categories completed (WCST-NC) given the number of times that 10 consecutive correct responses were made, reflecting overall success.

### Genotyping

Four polymorphisms spanning *DRD2* and *ANKK1* genes, and two polymorphisms encompassing *COMT* gene were selected on the basis of the previous association with schizophrenia, regarding linkage disequilibrium (LD) between SNPs and genes, genomic localization and minor allele frequencies in the Caucasian population provided by the international Hapmap Project or previously associated with psychiatric disorders. SNPs were genotyped using SNP Taqman assays. For quality control, 40 samples were genotyped in duplicate for all SNPs with 100% of concordance rate. The vulnerability allele or genotype to schizophrenia was made a priori from previous results of the literature.

### Statistical analysis

Demographic and clinical characteristics of samples were compared for continuous variables using an univariate analysis of variance (one way ANOVA) and categorical data were analysed using overall Chi-squared (χ^2^).

We estimated the pairwise linkage disequilibrium (LD) between all the markers by determining D’ and r^2^ from unaffected controls using the Haploview 4.1 program [49] (S1 Fig).

The association between SNPs and cognitive performances were tested by one-way ANOVA. First, genetic was the studied factor using the carrier of previous associated allele or genotype versus other, and cognitive performances were in the dependent list. Then, the two investigated factors were the genetics (carrier of previous associated allele or genotype versus other genotype or allele) and the clinical status (participants with schizophrenia versus healthy controls) that were combined to a total of 4 categories. The Levene’s test was carried out to test the homogeneity of the variances between groups. Each cognitive performance was included as dependent variables. Significant main effects of the genotypes were followed up separately in patients and controls.

To determine a significant interaction between genetics and disease on cognitive performances, all participants were included; carried variant (genotype or allele) previously associated to disease and diagnostic were treated as fixed effects in a mixed model (ANOVA by General Linear Model, SPSS, assuming Y = b0 + b1 x X1 + b2 x X2 + b3 x X1 x X2, where Y is the dependent variable, the cognitive test, X1 and X2 the fixed variable, respectively, disorder status and carried -or not- variant). In these analyses, the interaction of disorder and genetics is taking into account in the fitted model and the observed results report the association between the cognitive test and disorder status, carried variant and interaction. In the additive model, for each SNP, each allele was considered to have the same weight.

## Results

Demographic characteristics of the sample have been previously described [35]. Patients were younger than healthy controls (32.65 years (± 9.68) vs 43.77 years (± 12.18) p < 0.0001). There was a higher proportion of women in healthy controls than in patients (64% vs 31%, p < 0.0001). In participants with schizophrenia, the mean age at onset of the disease was 23.1 years (± 6.5), the mean duration of the illness was 7.2 years (± 3.4), the mean chlorpromazine equivalent daily dose was 323.46 mg (± 120.26). Patients had a lower level of education (college or higher education) than the control group (p = 0.0005).

We have previously shown that patients with schizophrenia performed significantly worse than controls in all cognitive performances, taking into account demographic variables: gender, educational level and age at interview [35]. The latency of selective attention (Stroop PI) was doubled in patients (33.5 ± 30.7) compared to controls (14.8 ± 4.9) [35]. The executive control (ANTc) and reaction time (ANT-RT) were significantly increased in patients (203.1 ± 110.3 and 807.5 ± 187.2) compared to controls (152.0 ± 37.0 and 705.0 ± 118.3) [35]. Patients completed less categories in WCST compared to controls (WCST-NC, 5.4 ± 1.1 vs 5.9 ± 0.5) and made more perseverative errors (WCST-PE, 16.7 ± 12.4 vs 8.7 ± 5.7) [35].

We examined the possible impact of carrying the vulnerability allele of 4 SNPs in the *DRD2*/*ANKK1* locus, and 2 SNPs of the *COMT* gene on cognitive performances in interaction with the disease status. The linkage disequilibrium was important among *DRD2* SNPs rs6265, rs6277 and rs2242592 with a D’ of 97 and r^2^ of 91 but not for rs1800497 (S1 Fig). For *COMT* SNPs, between rs4680 and rs165599, the D’ was of 71 and the r^2^ of 29 (S1 Fig). In control group, when we compared the cognitive performances according to the allele, genotype or carrier for each SNP, no significant difference was observed (data not shown). In the patients group, we observed differences in cognitive performances according to genetics. Thus, the performances of all cognitive tests were studied in patients group and also compared between patients and controls, carrying vulnerability allele or genotype versus not, for every SNP of each gene and reported in Tables 1 and 2 (also in S1 and S2 Tables).

**Table 1:** Association between genetic variants of *DRD2* genes and 6 cognitive scores (mean, SD) in 52 schizophrenia patients.

		STROOP PI	ANTc	ANT-RT	ANT%	WCST-NC	WCST-PE
**rs6275**	Carrier C (mean)	27.62	198.17	786.48	88.64	5.40	16.09
	Carrier C (SD)	17.43	105.57	173.74	14.78	1.06	11.44
	Carrier C (N)	42	42	42	42	42	42
	**Genotype TT** (mean)	63.25	217.75	907.12	85.37	5.28	16
	**Genotype TT** (SD)	61.72	146.76	248.01	14.42	1.25	11.03
	**Genotype TT** (N)	8	8	8	8	8	8
	F (df = 1)	10.47	0.20	2.82	0.33	0.07	0.01
	p	0.002	0.65	0.10	0.57	0.79	0.99
**rs6277**	**Carrier C** (mean)	30.92	203.97	808.22	87.64	5.59	14.28
	**Carrier C** (SD)	22.14	109.96	206.77	15.07	0.87	9.89
	**Carrier C** (N)	36	36	36	36	36	36
	Genotype TT (mean)	31.4	124.2	814.8	88.4	5.4	11.8
	Genotype TT (SD)	22.44	83.57	169.61	14.26	0.89	2.86
	Genotype TT (N)	5	5	5	5	5	5
	F (df = 1)	0.01	2.42	0.01	0.01	0.19	0.30
	p	0.96	0.13	0.95	0.92	0.66	0.58
**rs2242592**	Carrier T (mean)	28.25	202.57	796.00	88.25	5.38	16.6
	Carrier T (SD)	17.60	106.13	172.31	4.60	1.08	11.49
	Carrier T (N)	40	40	40	40	40	40
	**Genotype CC** (mean)	53.7	196.2	844.9	87.7	5.44	13.78
	**Genotype CC** (SD)	58.09	137.72	255.78	13.66	1.13	10.57
	**Genotype CC** (N)	10	10	10	10	9	9
	F (df = 1)	5.87	0.03	0.53	0.01	0.03	0.46
	p	0.019	0.87	0.47	0.92	0.86	0.50
**rs1800497**	**Carrier C** (A2) (mean)	31.61	194.41	801.43	87.89	5.37	15.79
	**Carrier C** (A2) (SD)	32.52	105.49	197.14	15.46	1.07	10.53
	**Carrier C** (N)	44	44	44	44	43	43
	Genotype TT (mean)	45.83	251.83	837.67	89.83	5.5	18.17
	Genotype TT (SD)	14.81	151.28	132.50	6.08	1.22	16.80
	Genotype TT (N)	6	6	6	6	6	6
	F (df = 1)	1.10	1.41	0.19	0.09	0.07	0.23
	p	0.30	0.24	0.67	0.76	0.79	0.63

ANTc: Attention Network Test executive control; ANT-RT: Attention Network Test median reaction time; ANT%: Attention Network Test percentage of correct answers;

WCST-NC: The Wisconsin Card Sorting Test number of categories completed; WCST-PE: The Wisconsin Card Sorting Test numbers of perseverative errors;

STROOP PI: Stroop Color-Word interference score; df: degree of freedom; In bold: vulnerability allele or genotype previously associated to schizophrenia;

Statistical analysis is a one-way ANOVA; p values of the one-way ANOVA analyses are indicated; SD: standard deviation; N: number.

**Table 2:** Association between genetic variants of *COMT* genes and 6 cognitive scores (mean, SD) in 52 schizophrenia patients.

		STROOP PI	ANTc	ANT-RT	ANT%	WCST-NC	WCST-PE
**rs4680**	Carrier A (Met) (mean)	28.86	191.68	809.32	88.86	5.61	12.75
	Carrier A (Met) (SD)	17.50	111.05	202.36	14.59	0.83	5.33
	Carrier A (Met) (N)	28	28	28	28	28	28
	**Genotype GG** (Val/Val) (mean)	35.54	199.77	808.38	85.31	5.46	16.61
	**Genotype GG** (Val/Val) (SD)	29.60	110.12	205.35	15.56	0.97	14.67
	**Genotype GG** (Val/Val) (N)	13	13	13	13	13	13
	F (df = 1)	0.82	0.05	0.01	0.50	0.25	1.54
	p	0.37	0.83	0.99	0.48	0.62	0.22
**rs165599**	**Carrier G** (mean)	33.92	211.42	830.69	84.77	5.54	15.19
	**Carrier G** (SD)	23.76	122.04	228.89	16.53	0.86	10.94
	**Carrier G** (N)	26	26	26	26	26	26
	Genotype AA (mean)	24.54	178.64	803.09	91.73	5.54	11.73
	Genotype AA (SD)	17.53	87.32	136.92	10.79	1.03	5.35
	Genotype AA (N)	11	11	11	11	11	11
	F (df = 1)	1.39	0.65	0.14	1.64	0.01	0.99
	p	0.25	0.43	0.71	0.21	0.98	0.33

ANTc: Attention Network Test executive control; ANT-RT: Attention Network Test median reaction time; ANT%: Attention Network Test percentage of correct answers;

WCST-NC: The Wisconsin Card Sorting Test number of categories completed; WCST-PE: The Wisconsin Card Sorting Test numbers of perseverative errors;

STROOP PI: Stroop Color-Word interference score; df: degree of freedom; In bold: vulnerability allele or genotype previously associated to schizophrenia;

Statistical analysis is a one-way ANOVA; p values of the one-way ANOVA analyses are indicated; SD: standard deviation; N: number.

### The *DRD2/ANKK1* SNPs impact on cognitive functions

**Fig 1:**
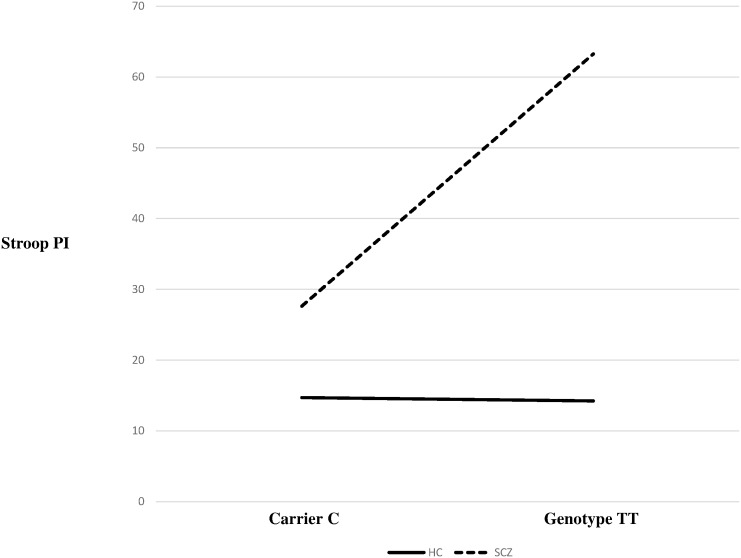
rs6275 association with Stroop-PI latency score according to carried variant in 52 patients with schizophrenia and 53 healthy controls.

The results of selective attention showed a significant impact of the carrier of the vulnerability allele or genotype of the four *DRD2/ANKK1* SNPs on Stroop-Interference effect. We found an association with worse performance in Stroop-PI and carriers of vulnerability TT genotype of rs6275 for schizophrenia patients compared to C carriers (63.65 ± 61.72 vs 27.62 ± 17.43, p = 0.002) (Table 1). The mean latencies at Stroop-PI interference score in the schizophrenia group were over two times higher than the control group for each of these vulnerability alleles or genotypes (S1 Table). According to the disease and the genetics, we observed a significant difference between patients and controls carrying the TT genotype versus the C allele (Fig 1). Among carriers of the vulnerability C allele of the rs6277, the score in patients was 30.92 ± 22.14 compared to 15 ± 4.98 in controls (p < 0.001) (S1 Table). For rs2242592, patients carrying the vulnerability CC genotype performed worse than patients without (53.7 ± 58.09 versus 28.23 ± 17.60, p = 0.019) (Table 1). Patients (31.61 ± 32.52) and healthy controls (14.23 ± 4.61) with the vulnerability C allele of rs1800497 performed better on the Stroop-PI latency than patients (45.83 ± 14.81) and healthy controls (21 ± 6.24) without (p = 0.0003) (S1 Table).

Impact on the Stroop-PI performance of the genotypes of rs6275 in *DRD2/ANKK1* in participants. Significant effects of diagnosis, genotypes and their interaction were showed. The genotype effect was observed in patients with schizophrenia, but not in healthy subjects. Patients with schizophrenia showed worse performance in Stroop-PI latency than healthy subjects. Patients carrying of vulnerability C allele showed lower performances than patients with TT genotype.

Performing ANT, patients harboring the vulnerability allele or genotype displayed a greater conflict effect score, a longer ANT-RT and a lower ANT%, as compared to controls (S1 Table).

When examining the WCST score, the number of perseverative errors was significantly different between genotype groups (S1 Table). In particular, patients carrying the vulnerability allele or genotype made significantly more perseverative errors than controls for the four SNPs (for example, rs6275 16± 11.03 vs 10.5± 8.19 p = 0.0009) (S1 Table).

### The *COMT* SNPs impact on cognitive functions

Homozygous patients genotyped Val/Val for rs4680 or carriers of G allele for rs165599 performed worse for the Stroop-PI than patients carrying Met or AA genotype for these SNPs but this was not significantly different (35.54 ±29.60 vs 28.86 ± 17.50, p = 0.37 and 33.92 ±23.76 vs 24.54 ± 17.53, p = 0.25, respectively) (Table 2). No difference was observed in healthy controls according to the carried allele. The main effect of genotypes on selective attention was significant for the Stroop-PI in patients compared to controls (S2 Table).

Performance in conflict effect of attention was not statistically significant between groups. However, the ANT-RT was significantly longer and the ANT% was significantly less marked in patients carrying the vulnerability allele or genotype of rs4680 compared to control subjects (p = 0.009 and p = 0.01, respectively). For rs165599, the ANT-RT was also significantly longer and the ANT% significantly lower when subjects carried the G allele as compared with AA homozygotes between groups (p = 0.01 and p = 0.006, respectively) (S2 Table).

For WCST, homozygous patients for the Val allele of rs4680 made more perseverative errors (16. 61 ± 14.67) than patients carried Met allele (12.75 ± 5.33) but this was not significantly different (p = 0.22) (Table 2). For rs165599, patients carried G allele made more perseverative errors than subjects genotyped AA (15.19± 10.94 vs 11.73± 5.35 p = 0.33). Genetics of the controls do not influence their performances (S2 Table). The main effect of genetics for WCST was significant only for the WCST-PE between groups of patients and controls (S2 Table).

### Disease by genetics interactions

Because we observed major effects according to the disease status and some effect regarding the allele or genotype of vulnerability in patients on cognitive performances, we also investigated possible interactions between genetics and disease on cognitive performances. We observed a significant interaction between disease and genetics for rs6275 and rs2242592 only for the Stroop-PI performance (p = 0.002 and p = 0.024, respectively) (Table 3 and S3 Table). Furthermore, a significant effect of genetics was found for rs6275 and rs2242592 in the *DRD2* gene, only on the Stroop-PI (S3 Table). Considering an additive model where the effects of each allele were treated as equal, we found significant genetic effects and disorder by genetics interactions (S4 Table). The polymorphisms of rs6275, rs2242592 and rs1800497 of the *DRD2* gene showed an additive influence on ANT% (p = 0.018, p = 0.017, p = 0.014, respectively) and the disorder by genetics interactions were also significant for these SNPs (p = 0.02, p = 0.022, p = 0.017, respectively). In the same way, rs6277 of the *DRD2* gene, rs4680 and rs165599 of the *COMT* gene showed an additive influence on the WCST-NC (p < 0.001, p < 0.001, p = 0.019, respectively) and the WCST-PE (p < 0.001, p < 0.001, p = 0.001, respectively). A disorder by genetics interaction was also significant for rs6277, rs4680 and rs165599 explaining performance on the WCST-NC (p < 0.001, p < 0.001, p = 0.015, respectively) and the WCST- PE (p < 0.001, p < 0.001, p = 0.01, respectively).

**Table 3:** Disorder and genetics interaction on genetic data for cognitive performances to STROOP-PI.

SNP	Gene	Carried Allele (N HC vs N SCZ)	Carried Genotype (N HC vs N SCZ)	Disorder X Genetics	
				F	p Value
rs6275	DRD2	C (43 vs 42)	TT (8 vs 8)	***10*.*54***	***0*.*002***
rs6277	DRD2	C (23 vs 36)	TT (8 vs 5)	.12	0.73
rs2242592	DRD2	T (44 vs 40)	CC (7 vs 10)	***5*.*25***	***0*.*024***
rs1800497	DRD2	C (48 vs 44)	TT (3 vs 6)	.21	0.65
rs4680	COMT	Met (23 vs 28)	Val/Val (7 vs 13)	.53	0.47
rs165599	COMT	G (13 vs 26)	AA (16 vs 11)	2.09	0.15

STROOP PI: Stroop Color-Word interference score; SNP: single nucleotide polymorphism

Statistical analysis of Disorder X Genetics is extracted from the fitted model of GLM two-way ANOVA and the observe results report the association between the cognitive test and interaction Disorder X Genetics. P values are for the interaction Disorder X Genetics only from the two-way ANOVA

## Discussion

### *DRD2/ANKK1* gene, cognitive tasks and schizophrenia

The main finding of this exploratory study involves rs6275 and rs2242592 of the *DRD2/ANKK1* locus and Stroop Color-Word in schizophrenia. The worst Stroop-PI results were observed in patients with the vulnerability genotype of rs6275 and rs22422592. Interestingly, due to the multiple analyses performed in this study, we carried out a Bonferroni correction, implementing the 6 SNPs and the 6 measures of tests. Corrected p values for rs6275 and rs22422592 remained significant (respectively 0.000004 and 0.0002). These findings suggested that the TT genotype of rs6275 and CC genotype of rs22422592 are associated with a marked deterioration of selective attention and that this effect is more important in participants with schizophrenia. We showed an interaction between schizophrenia and the genetic effect of rs6275 and rs22422592 on Stroop-PI, but the effect of the disease appears to be more prominent.

Patients harboring the vulnerability TT genotype of rs6275 were less efficient than controls on the executive control of the Attention Network Test scores (ANTc, ANT-RT and ANT%). They took a longer time to resolve conflict and showed a lower percentage of correct answers than control subjects. We previously found that the ANT-RT may be a possible endophenotype marker for schizophrenia [35]. Considering an additive model where the two alleles have the same influence, we found only a significant illness by genetics interaction influencing the percentage of correct answers (ANT%) reinforcing the potential role of the TT genotype instead of carrier of one T allele only. In this study, rs6275 SNPs interacted with schizophrenia on attention performances. Carrying the vulnerability TT genotype of rs6275 is associated with a decline in executive functions compared to controls as indicated also by the great number of perseverative errors of WCST.

All together, these data suggest that rs6275 affects executive and attentional performance, specifically in schizophrenia. This is consistent with previous studies supporting the association between rs6275 and schizophrenia [50, 51]. Although the association between rs6275 and susceptibility to schizophrenia is documented, this research is the first, to our knowledge, to investigate their interaction with attention and executive functions.

Contrary to healthy controls, patients with schizophrenia who carry the vulnerability C allele of rs6277 perform worse in perseverative errors than patients without (Table 1). The current results are in line with a previous study in healthy volunteers [36]. Regarding executive functions, the C allele of rs6277 modulates this effect according to illness status. Considering rs1800497 of the *DRD2/ANKK1* locus, impaired selective attention (assessed by Stroop-PI) is associated with schizophrenia more than genetics. Using the additive model, we found a significant interaction between schizophrenia and the C allele of rs1800497 on the percentage of correct answers (ANT%). Carrying this allele increases conflict especially in schizophrenia patients. We had previously found that SNPs rs1800497, rs2242592 and rs6277 were significantly associated with susceptibility for schizophrenia [7]. Each of them has probably no direct effect on attention but they act via the disease status.

### *COMT* gene, cognitive tasks and schizophrenia

In this work, patients have scored significantly worse on neuropsychological tests of executive cognition, but we have failed to find any association with the *COMT* polymorphisms when the vulnerability alleles were considered as dominant (genotype Val/Val for rs4680 and G allele for rs165599). The additive pattern raises the sample size and enhances the significance.

For rs4680 and the number of perseverative errors, there is a discrepancy between patients and healthy controls. Contrary to healthy controls, patients carrying the Val/Val genotype have performed worse than the carriers of Met allele. We found that higher loading of the COMT 158 Met allele was associated with better neurocognitive performance among individuals with chronic schizophrenia, consistent with and extending the findings from prior studies [28, 52]. Bilder et al. [52] did not find any association between the homozygous patients Val/Val and the executive function (WCST), but a significant genotype effect was observed on Processing Speed and Attention. Moreover, in this last study, the sample size was relatively small, participants had a long duration of illness (more than 17 years), and no healthy controls was included. Egan and colleagues have display that Val/Val subject have the poorest performance [28]. Disease status seems to modulate the action of *COMT* genotype on executive cognition.

For rs165599, the G allele is associated with worse STROOP-PI performances and a decrease level of performance on the perseverative errors of executive cognition. This effect could be link with schizophrenia.

In current sample, *COMT* genotypes are associated with executive cognition and this effect is related to schizophrenia diagnosis. Observed effects of *COMT* Val158Met genotype showed either a relative advantage or disadvantage on cognitive functions, in healthy controls and in diseases (for review [53]). These effects are dependent on the dopaminergic neurotransmission charge in subjects. Thus, the Met allele reduces the dopamine degradation which presents a benefit, compared to Val allele, for cognitive function in healthy controls and in schizophrenic patients (for review [53]).

We did not replicate the previously observed association of *COMT* SNPs with executive control of attention. Our results are in contradiction with those of Opgen-Rhein et al. [54] who have reported reduced conflict effect in a group of schizophrenia patients where the Met allele carriers showed significantly higher frequency. It should be mentioned that this last work included only male participants, with a greater mean RT, which is associated with a higher conflict effect. This may explain the discrepancy in the results.

The main strengths of the present study are: (1) the homogeneity of the clinical characteristics of participants with schizophrenia which is an important criteria for genetic studies of complex disorders, (2) the short duration of illness and (3) the treatment with antipsychotic medications known to preserve cognitive functions. Furthermore, a large number of cognitive tests have been used and our results are in line with the literature. This homogenous group of outpatients with schizophrenia displayed an overall deficit compared to controls in executive functions measured using the ANT, WCST and Stroop tests after adjusting for demographic characteristic (i.e. sex ratio, years of education age at interview). This suggested that the deficits observed in patients with schizophrenia could be attributed directly to the illness, rather than to these variables that might affect cognition performances. The limitation of this work is the sample size, which may affect the statistical power to detect minor effect especially for genetics. However, our size of sample for patients and healthy controls is higher in this work compared to the previous studies of cognitive tasks in schizophrenia and we included the genetic analysis. Furthermore, a larger cohort would be useful to analyze the effect of carrying one or two vulnerability alleles. Then, our results need to be replicated in a greater sample.

## Conclusions

In our sample, schizophrenia and genetics interact on the executive control of attention, mostly on Stroop-PI and slightly on ANT. Rs6275, rs2242592 and rs1800497 of the *DRD2* increased the conflict while *COMT* SNPs do not. Regarding executive cognition, the *COMT* and schizophrenia also interact.

The anterior cingulate cortex is activated by the conflict effect [24] and by the selective attention [55]. The executive component of cognition is related to dorsolateral prefrontal cortex. This is in accordance with Egan and colleagues [28] who suggest an impaired prefrontal cognition in schizophrenia. These results suggest that the *DRD2* SNPs may modulate the anterior cingulate cortex function while the *COMT* SNPs may have neurobiological effects specific to the dorsolateral prefrontal cortex.

## Supporting Information

S1 Fig Pairwise linkage disequilibrium of SNPs among *COMT* and *DRD2* genes. (TIF)

S1 Table Association between genetic variants of *DRD2* genes and 6 cognitive scores (mean, SD) in 52 schizophrenia patients and 53 healthy controls. (XLSX)

S2 Table Association between genetic variants of *COMT* genes and 6 cognitive scores (mean, SD) in 52 schizophrenia patients and 53 healthy controls. (XLSX)

S3 Table General linear model for association between cognitive tests and disorder, genetics and their interaction effects. (XLSX)

S4 Table Disorder, genetics and their interaction in additive model. (XLSX)
