# Supplementary figures and images for: Impact of DRD2/ANKK1 and COMT Polymorphisms on Attention and Cognitive Functions in Schizophrenia
Source: PLoS One. 2017 Jan 13;12(1):e0170147. doi: 10.1371/journal.pone.0170147 (PMC5235377; doi:10.1371/journal.pone.0170147)

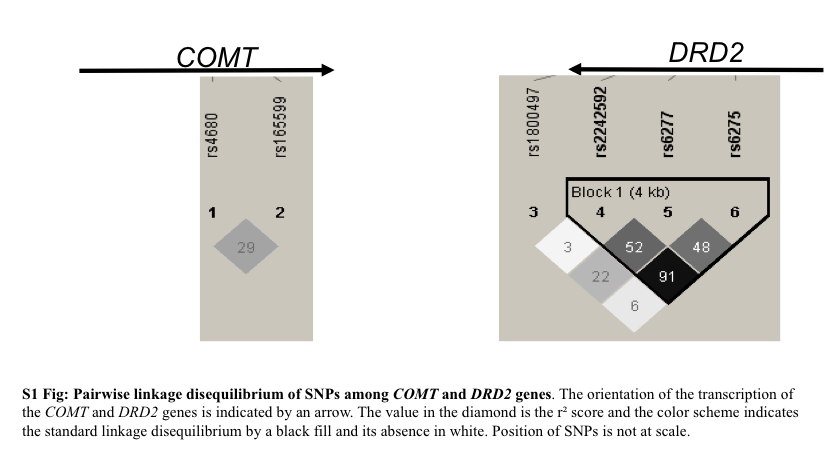

Supplement: S1 Fig — (TIF) [file pone.0170147.s001.tif]
